# 5-({3-[(5-Amino-1,3,4-thia­diazol-2-yl)sulfanylmeth­yl]benz­yl}sulfan­yl)-1,3,4-thia­diazol-2-amine

**DOI:** 10.1107/S1600536812013116

**Published:** 2012-03-31

**Authors:** Sung Kwon Kang, Nam Sook Cho, Siyoung Jang

**Affiliations:** a Department of Chemistry, Chungnam National University, Daejeon 305-764, Republic of Korea

## Abstract

In the title compound, C_12_H_12_N_6_S_4_, the two terminal thia­diazole rings are twisted with respect to the central benzene ring, making dihedral angles of 54.28 (4) and 76.56 (3)°. The dihedral angle between the two thia­diazole rings is 27.77 (4)°. Inter­molecular N—H⋯N hydrogen bonds stabilize the crystal packing, linking the mol­ecules into a tape along the *b* axis.

## Related literature

For the synthesis and reactivity of thia­diazole derivatives, see: Cho *et al.* (1993 ▶, 2001 ▶) and for the synthesis and reactivity of macrocyclic compounds with thia­diazole derivatives, see: Cho *et al.* (2002 ▶, 2006 ▶). For related structures of thia­diazole derivatives, see: Kang, Cho & Jang (2012 ▶); Kang, Cho & Jeon (2012 ▶).
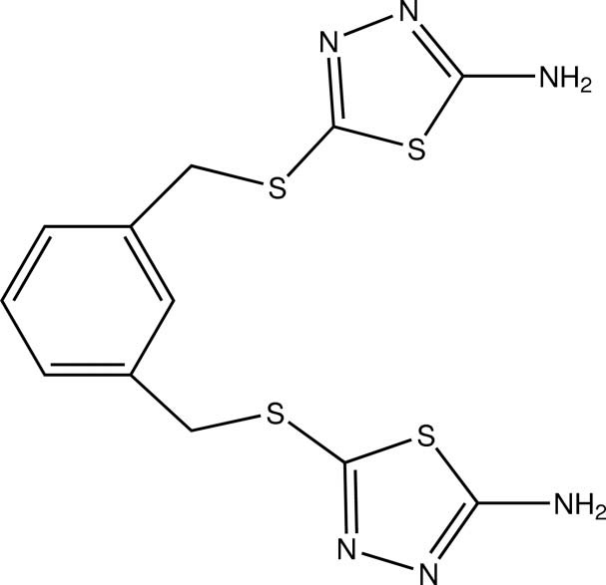


## Experimental

### 

#### Crystal data

C_12_H_12_N_6_S_4_
*M* *_r_* = 368.52
Monoclinic, 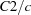

*a* = 16.3579 (10) Å
*b* = 6.1382 (4) Å
*c* = 30.7095 (18) Å
β = 90.373 (1)°
*V* = 3083.4 (3) Å^3^
*Z* = 8
Mo *K*α radiation
μ = 0.62 mm^−1^
*T* = 296 K
0.14 × 0.12 × 0.06 mm

#### Data collection

Bruker SMART CCD area-detector diffractometer
Absorption correction: multi-scan (*SADABS*; Bruker, 2002 ▶) *T*_min_ = 0.91, *T*_max_ = 0.96
22777 measured reflections
3836 independent reflections
2387 reflections with *I* > 2σ(*I*)
*R*_int_ = 0.082

#### Refinement

*R*[*F* ^2^ > 2σ(*F* ^2^)] = 0.039
*wR*(*F* ^2^) = 0.077
*S* = 0.88
3836 reflections
199 parameters
H-atom parameters constrained
Δρ_max_ = 0.27 e Å^−3^
Δρ_min_ = −0.35 e Å^−3^

### 

Data collection: *SMART* (Bruker, 2002 ▶); cell refinement: *SAINT* (Bruker, 2002 ▶); data reduction: *SAINT*; program(s) used to solve structure: *SHELXS97* (Sheldrick, 2008 ▶); program(s) used to refine structure: *SHELXL97* (Sheldrick, 2008 ▶); molecular graphics: *ORTEP-3* (Farrugia, 1997 ▶); software used to prepare material for publication: *WinGX* (Farrugia, 1999 ▶).

## Supplementary Material

Crystal structure: contains datablock(s) global, I. DOI: 10.1107/S1600536812013116/is5100sup1.cif

Structure factors: contains datablock(s) I. DOI: 10.1107/S1600536812013116/is5100Isup2.hkl

Supplementary material file. DOI: 10.1107/S1600536812013116/is5100Isup3.cml

Additional supplementary materials:  crystallographic information; 3D view; checkCIF report

## Figures and Tables

**Table 1:** Hydrogen-bond geometry (Å, °)

*D*—H⋯*A*	*D*—H	H⋯*A*	*D*⋯*A*	*D*—H⋯*A*
N14—H14*A*⋯N20^i^	0.86	2.27	3.005 (3)	144
N14—H14*B*⋯N13^ii^	0.86	2.31	3.142 (2)	162
N22—H22*A*⋯N12^iii^	0.86	2.15	3.007 (3)	171
N22—H22*B*⋯N21^iv^	0.86	2.14	2.982 (3)	168

Symmetry codes: (i) 
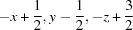
; (ii) 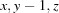; (iii) 
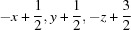
; (iv) 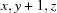.

**Fig. 1.**
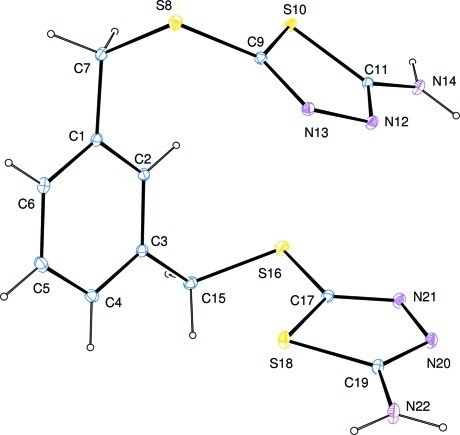
Molecular structure of the title compound, showing the atom-numbering scheme and 30% probability ellipsoids.

**Fig. 2.**
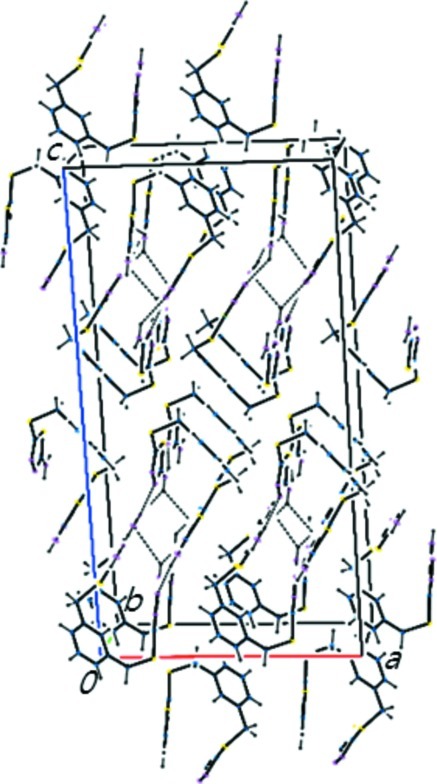
Part of the crystal structure of the title compound, showing molecules linked by intermolecular N—H···N hydrogen bonds (dashed lines).

## supplementary crystallographic information

### Comment

Polydentate macrocyclic compounds containing heterocyclic rings as a subunit possessed a variety of interesting properties. The 5-amino-3*H*-1,3,4-thiadizoline-2-thione has received attention as s sulfur donor subunit (Cho *et al.*, 1993, 2001). Thus, we reported novel macrocycles that incorporated 5-amino-3*H*-1,3,4-thiadizoline-2-thiones (Cho *et al.*, 2002, 2006). The title compound is an intermediate to prepare the macrocyclic compounds with the ring closure reaction of two terminal amino groups.

Two five-membered 1,3,4-thiadiazol-2-yl units are planar, with r.m.s. deviations of 0.015 and 0.024 Å from the corresponding squares plane defined by the seven constituent atoms. The bond distances of C9—N13 and C11—N12 [1.294 (2) and 1.316 (3) Å]; C17—N21 and C19—N20 [1.285 (3) and 1.306 (3) Å] in two thiadiazole rings (S8—N14 atoms and S16—N22 atoms) are comparable with those of other thiadiazole compounds for double bond character (Kang, Cho & Jang, 2012; Kang, Cho & Jeon, 2012). The dihedral angles between *m*-xylene and two thiadiazole rings are 54.28 (4) and 76.56 (3)° (Fig. 1). Two terminal thiadiazole rings are not parallel, with a dihedral angle of 27.77 (4)°. The crystal structure is stabilized by the intermolecular N—H···N hydrogen bonds, which link the molecules into one-dimensional chains along the *b*-axis (Table 1 and Fig. 2).

### Experimental

α,α'-Dibromoxylene (1.5 g, 5.8 mmol) was added to a solution of 5-amino-3*H*-1,3,4-thiadizoline-2-thione (1.4 g, 10.6 mmol) dissolved in EtOH (50 ml)-KOH (0.63 g, 11.2 mmol). The resulting mixture was heated under reflux until the reactant was disappeared on TLC. The solvent was evaporated under reduced pressure to leave a solid residue, which was washed with water. The crude product was recrystallized from methanol (product yield 93%). Colourless crystals of (I) were obtained from its DMSO solution by slow evaporation of the solvent at room temperature, m.p. 202–205 °C, *R*_f_, 0.13 (n-hexane: EA: ethanol = 5: 3: 1 *v*/*v*). ^1^H NMR (DMSO-d_6_, p.p.m.) 7.35 (b, 4*H*, 2NH_2_), 7.27–7.23 (m, 4*H*, C_6_H_4_), 4.29 (s, 4*H*, 2CH_2_); ^13^C NMR (DMSO-d_6_, p.p.m.): 169.9 (C=N), 149.4 (C—S), 137.3, 129.5, 128.6, 128.1 (C_6_H_4_), 38.3 (SCH_2_). FABHRMS Cald. for C_12_H_12_N_6_S_4_ 369.0085; found 369.0082

### Refinement

H atoms were positioned geometrically and refined using a riding model, with C—H = 0.93 or 0.97 Å and N—H = 0.86 Å, and with *U*_iso_(H) = 1.2*U*_eq_(carrier C or N).

### Crystal data

**Table:**

C_12_H_12_N_6_S_4_	*F*(000) = 1520
*M**_r_* = 368.52	*D*_x_ = 1.588 Mg m^−^^3^
Monoclinic, *C*2/*c*	Mo *K*α radiation, λ = 0.71073 Å
Hall symbol: -C 2yc	Cell parameters from 3247 reflections
*a* = 16.3579 (10) Å	θ = 2.5–22.1°
*b* = 6.1382 (4) Å	µ = 0.62 mm^−^^1^
*c* = 30.7095 (18) Å	*T* = 296 K
β = 90.373 (1)°	Block, colourless
*V* = 3083.4 (3) Å^3^	0.14 × 0.12 × 0.06 mm
*Z* = 8	

### Data collection

**Table:**

Bruker SMART CCD area-detector diffractometer	2387 reflections with *I* > 2σ(*I*)
Graphite monochromator	*R*_int_ = 0.082
φ and ω scans	θ_max_ = 28.3°, θ_min_ = 1.3°
Absorption correction: multi-scan (*SADABS*; Bruker, 2002)	*h* = −21→21
*T*_min_ = 0.91, *T*_max_ = 0.96	*k* = −8→8
22777 measured reflections	*l* = −40→40
3836 independent reflections	

### Refinement

**Table:**

Refinement on *F*^2^	Primary atom site location: structure-invariant direct methods
Least-squares matrix: full	Secondary atom site location: difference Fourier map
*R*[*F*^2^ > 2σ(*F*^2^)] = 0.039	Hydrogen site location: inferred from neighbouring sites
*wR*(*F*^2^) = 0.077	H-atom parameters constrained
*S* = 0.88	*w* = 1/[σ^2^(*F*_o_^2^) + (0.028*P*)^2^] where *P* = (*F*_o_^2^ + 2*F*_c_^2^)/3
3836 reflections	(Δ/σ)_max_ = 0.001
199 parameters	Δρ_max_ = 0.27 e Å^−^^3^
0 restraints	Δρ_min_ = −0.35 e Å^−^^3^

### Special details

**Table:**

Geometry. All s.u.'s (except the s.u. in the dihedral angle between two l.s. planes) are estimated using the full covariance matrix. The cell s.u.'s are taken into account individually in the estimation of s.u.'s in distances, angles and torsion angles; correlations between s.u.'s in cell parameters are only used when they are defined by crystal symmetry. An approximate (isotropic) treatment of cell s.u.'s is used for estimating s.u.'s involving l.s. planes.
Refinement. Refinement of *F*^2^ against ALL reflections. The weighted *R*-factor *wR* and goodness of fit *S* are based on *F*^2^, conventional *R*-factors *R* are based on *F*, with *F* set to zero for negative *F*^2^. The threshold expression of *F*^2^ > 2σ(*F*^2^) is used only for calculating *R*-factors(gt) *etc*. and is not relevant to the choice of reflections for refinement. *R*-factors based on *F*^2^ are statistically about twice as large as those based on *F*, and *R*- factors based on ALL data will be even larger.

### Fractional atomic coordinates and isotropic or equivalent isotropic displacement parameters (Å^2^)

**Table:**

	*x*	*y*	*z*	*U*_iso_*/*U*_eq_	
C1	0.07088 (13)	0.5674 (4)	0.54356 (7)	0.0349 (5)	
C2	0.04110 (13)	0.4333 (3)	0.57612 (7)	0.0355 (5)	
H2	0.0609	0.2917	0.5784	0.043*	
C3	−0.01744 (12)	0.5042 (4)	0.60545 (7)	0.0354 (5)	
C4	−0.04693 (13)	0.7144 (4)	0.60143 (8)	0.0427 (6)	
H4	−0.0857	0.7658	0.6209	0.051*	
C5	−0.01915 (14)	0.8485 (4)	0.56860 (8)	0.0462 (6)	
H5	−0.0406	0.9881	0.5657	0.055*	
C6	0.04010 (14)	0.7777 (4)	0.54005 (7)	0.0428 (6)	
H6	0.0594	0.8706	0.5185	0.051*	
C7	0.13626 (13)	0.4864 (4)	0.51346 (7)	0.0400 (5)	
H7A	0.1306	0.5602	0.4857	0.048*	
H7B	0.128	0.332	0.5084	0.048*	
S8	0.24002 (3)	0.52863 (9)	0.534089 (19)	0.03947 (16)	
C9	0.24017 (12)	0.3680 (3)	0.58115 (7)	0.0327 (5)	
S10	0.22830 (4)	0.08693 (9)	0.580246 (18)	0.03689 (15)	
C11	0.24370 (12)	0.0940 (4)	0.63625 (7)	0.0324 (5)	
N12	0.25717 (11)	0.2911 (3)	0.65155 (6)	0.0376 (4)	
N13	0.25390 (10)	0.4480 (3)	0.61950 (6)	0.0357 (4)	
N14	0.24306 (11)	−0.0849 (3)	0.66089 (6)	0.0426 (5)	
H14A	0.2518	−0.0752	0.6885	0.051*	
H14B	0.2339	−0.21	0.6492	0.051*	
C15	−0.04979 (13)	0.3540 (4)	0.64040 (7)	0.0430 (6)	
H15A	−0.0909	0.2599	0.6274	0.052*	
H15B	−0.0766	0.4418	0.6624	0.052*	
S16	0.02620 (4)	0.18512 (10)	0.66701 (2)	0.04862 (18)	
C17	0.07177 (13)	0.3682 (3)	0.70332 (7)	0.0363 (5)	
S18	0.06869 (4)	0.64865 (10)	0.69824 (2)	0.04757 (18)	
C19	0.12945 (13)	0.6580 (4)	0.74495 (7)	0.0391 (5)	
N20	0.14884 (12)	0.4662 (3)	0.76028 (6)	0.0430 (5)	
N21	0.11434 (12)	0.2993 (3)	0.73582 (6)	0.0455 (5)	
N22	0.15219 (12)	0.8447 (3)	0.76364 (6)	0.0564 (6)	
H22A	0.1812	0.8428	0.7871	0.068*	
H22B	0.1378	0.9669	0.7522	0.068*	

### Atomic displacement parameters (Å^2^)

**Table:**

	*U*^11^	*U*^22^	*U*^33^	*U*^12^	*U*^13^	*U*^23^
C1	0.0349 (12)	0.0417 (14)	0.0280 (12)	−0.0008 (10)	−0.0064 (10)	−0.0027 (10)
C2	0.0362 (12)	0.0349 (13)	0.0352 (13)	0.0035 (10)	−0.0030 (10)	−0.0004 (10)
C3	0.0317 (12)	0.0423 (14)	0.0321 (12)	−0.0024 (10)	−0.0065 (10)	−0.0041 (10)
C4	0.0346 (13)	0.0476 (15)	0.0459 (15)	0.0054 (11)	−0.0044 (11)	−0.0113 (12)
C5	0.0479 (14)	0.0370 (14)	0.0536 (16)	0.0071 (11)	−0.0094 (13)	−0.0018 (12)
C6	0.0462 (14)	0.0424 (15)	0.0396 (14)	−0.0021 (11)	−0.0070 (12)	0.0072 (11)
C7	0.0456 (13)	0.0470 (14)	0.0274 (12)	−0.0006 (11)	−0.0020 (10)	0.0012 (10)
S8	0.0410 (3)	0.0396 (3)	0.0378 (3)	−0.0032 (3)	0.0021 (3)	0.0052 (3)
C9	0.0325 (12)	0.0314 (12)	0.0342 (12)	0.0007 (9)	−0.0004 (10)	−0.0016 (10)
S10	0.0477 (3)	0.0329 (3)	0.0300 (3)	−0.0021 (3)	−0.0028 (3)	−0.0039 (2)
C11	0.0291 (11)	0.0390 (13)	0.0293 (12)	0.0001 (10)	−0.0014 (9)	0.0001 (10)
N12	0.0464 (11)	0.0360 (11)	0.0303 (10)	−0.0010 (9)	−0.0045 (9)	−0.0034 (8)
N13	0.0402 (11)	0.0322 (11)	0.0346 (11)	0.0009 (8)	−0.0033 (9)	−0.0035 (8)
N14	0.0574 (13)	0.0386 (11)	0.0317 (11)	−0.0075 (10)	−0.0053 (9)	0.0026 (9)
C15	0.0394 (13)	0.0529 (15)	0.0368 (13)	−0.0049 (11)	0.0008 (11)	−0.0068 (11)
S16	0.0673 (4)	0.0363 (4)	0.0422 (4)	−0.0041 (3)	−0.0036 (3)	0.0014 (3)
C17	0.0404 (13)	0.0365 (13)	0.0320 (13)	−0.0004 (10)	0.0039 (10)	0.0041 (10)
S18	0.0620 (4)	0.0349 (3)	0.0455 (4)	−0.0020 (3)	−0.0187 (3)	0.0072 (3)
C19	0.0411 (13)	0.0430 (15)	0.0331 (13)	−0.0007 (11)	−0.0043 (10)	0.0065 (11)
N20	0.0550 (13)	0.0369 (12)	0.0370 (11)	0.0018 (10)	−0.0075 (10)	0.0058 (9)
N21	0.0605 (13)	0.0370 (12)	0.0389 (12)	0.0034 (10)	−0.0054 (10)	0.0045 (9)
N22	0.0771 (16)	0.0392 (12)	0.0525 (14)	−0.0017 (11)	−0.0322 (12)	0.0058 (10)

### Geometric parameters (Å, º)

**Table:**

C1—C2	1.386 (3)	S10—C11	1.737 (2)
C1—C6	1.390 (3)	C11—N12	1.316 (3)
C1—C7	1.503 (3)	C11—N14	1.334 (3)
C2—C3	1.389 (3)	N12—N13	1.378 (2)
C2—H2	0.93	N14—H14A	0.86
C3—C4	1.383 (3)	N14—H14B	0.86
C3—C15	1.513 (3)	C15—S16	1.810 (2)
C4—C5	1.381 (3)	C15—H15A	0.97
C4—H4	0.93	C15—H15B	0.97
C5—C6	1.381 (3)	S16—C17	1.747 (2)
C5—H5	0.93	C17—N21	1.285 (3)
C6—H6	0.93	C17—S18	1.729 (2)
C7—S8	1.826 (2)	S18—C19	1.741 (2)
C7—H7A	0.97	C19—N20	1.306 (3)
C7—H7B	0.97	C19—N22	1.333 (3)
S8—C9	1.749 (2)	N20—N21	1.388 (2)
C9—N13	1.294 (2)	N22—H22A	0.86
C9—S10	1.737 (2)	N22—H22B	0.86
			
C2—C1—C6	118.6 (2)	C9—S10—C11	86.78 (10)
C2—C1—C7	120.16 (19)	N12—C11—N14	123.81 (19)
C6—C1—C7	121.2 (2)	N12—C11—S10	113.52 (16)
C1—C2—C3	121.9 (2)	N14—C11—S10	122.66 (17)
C1—C2—H2	119	C11—N12—N13	112.45 (17)
C3—C2—H2	119	C9—N13—N12	113.00 (17)
C4—C3—C2	118.4 (2)	C11—N14—H14A	120
C4—C3—C15	120.6 (2)	C11—N14—H14B	120
C2—C3—C15	121.0 (2)	H14A—N14—H14B	120
C5—C4—C3	120.3 (2)	C3—C15—S16	115.29 (15)
C5—C4—H4	119.8	C3—C15—H15A	108.5
C3—C4—H4	119.8	S16—C15—H15A	108.5
C4—C5—C6	120.8 (2)	C3—C15—H15B	108.5
C4—C5—H5	119.6	S16—C15—H15B	108.5
C6—C5—H5	119.6	H15A—C15—H15B	107.5
C5—C6—C1	119.9 (2)	C17—S16—C15	102.05 (11)
C5—C6—H6	120	N21—C17—S18	114.41 (17)
C1—C6—H6	120	N21—C17—S16	120.73 (17)
C1—C7—S8	113.78 (15)	S18—C17—S16	124.80 (13)
C1—C7—H7A	108.8	C17—S18—C19	86.69 (11)
S8—C7—H7A	108.8	N20—C19—N22	123.6 (2)
C1—C7—H7B	108.8	N20—C19—S18	113.79 (17)
S8—C7—H7B	108.8	N22—C19—S18	122.63 (17)
H7A—C7—H7B	107.7	C19—N20—N21	111.92 (18)
C9—S8—C7	101.71 (10)	C17—N21—N20	113.17 (18)
N13—C9—S10	114.22 (16)	C19—N22—H22A	120
N13—C9—S8	122.50 (16)	C19—N22—H22B	120
S10—C9—S8	123.17 (12)	H22A—N22—H22B	120

### Hydrogen-bond geometry (Å, º)

**Table:**

*D*—H···*A*	*D*—H	H···*A*	*D*···*A*	*D*—H···*A*
N14—H14*A*···N20^i^	0.86	2.27	3.005 (3)	144
N14—H14*B*···N13^ii^	0.86	2.31	3.142 (2)	162
N22—H22*A*···N12^iii^	0.86	2.15	3.007 (3)	171
N22—H22*B*···N21^iv^	0.86	2.14	2.982 (3)	168

Symmetry codes: (i) −*x*+1/2, *y*−1/2, −*z*+3/2; (ii) *x*, *y*−1, *z*; (iii) −*x*+1/2, *y*+1/2, −*z*+3/2; (iv) *x*, *y*+1, *z*.
